# Assessing research impact in academic clinical medicine: a study using Research Excellence Framework pilot impact indicators

**DOI:** 10.1186/1472-6963-12-478

**Published:** 2012-12-23

**Authors:** Pavel V Ovseiko, Alis Oancea, Alastair M Buchan

**Affiliations:** 1 Medical Sciences Division, University of Oxford, Oxford, UK; 2 Department of Education, University of Oxford, Oxford, UK

## Abstract

**Background:**

Funders of medical research the world over are increasingly seeking, in research assessment, to complement traditional output measures of scientific publications with more outcome-based indicators of societal and economic impact. In the United Kingdom, the Higher Education Funding Council for England (HEFCE) developed proposals for the Research Excellence Framework (REF) to allocate public research funding to higher education institutions, *inter alia*, on the basis of the social and economic impact of their research. In 2010, it conducted a pilot exercise to test these proposals and refine impact indicators and criteria.

**Methods:**

The impact indicators proposed in the 2010 REF impact pilot exercise are critically reviewed and appraised using insights from the relevant literature and empirical data collected for the University of Oxford’s REF pilot submission in clinical medicine. The empirical data were gathered from existing administrative sources and an online administrative survey carried out by the university’s Medical Sciences Division among 289 clinical medicine faculty members (48.1% response rate).

**Results:**

The feasibility and scope of measuring research impact in clinical medicine in a given university are assessed. Twenty impact indicators from seven categories proposed by HEFCE are presented; their strengths and limitations are discussed using insights from the relevant biomedical and research policy literature.

**Conclusions:**

While the 2010 pilot exercise has confirmed that the majority of the proposed indicators have some validity, there are significant challenges in operationalising and measuring these indicators reliably, as well as in comparing evidence of research impact across different cases in a standardised manner. It is suggested that the public funding agencies, medical research charities, universities, and the wider medical research community work together to develop more robust methodologies for capturing and describing impact, including more valid and reliable impact indicators.

## Background

In the United Kingdom, universities and other higher education institutions (HEIs) conduct more than £1.3 billion worth of research in clinical medicine annually, most of which is funded by United Kingdom (UK) and European Union (EU) government agencies as well as medical research charities. Owing to the support of the public and taxpayers, the UK has developed some of the strongest and most productive clinical medicine research bases in the world. According to Thompson Reuters (ISI) bibliometric indicators, the UK’s clinical sciences research is second only to the USA. With just 0.9% of the world’s population, the UK produces 8.7% of world publications in clinical sciences and generates 12.7% of world citations [1].

Increasingly, however, funders of medical research the world over are seeking, in research assessment, to complement traditional output measures of scientific publications – such as number of publications, number of citations, impact factor, research funding, degree of co-authorship, and *h*-index [2,3] – with more outcome-based indicators of societal and economic impact [4-15]. The medical research and academic community is wondering how to measure returns on investment in health research [16,17], how to “best report to taxpayers and philanthropists on the societal value produced by the monies entrusted to [it]” [18], and how key indicators in academic medicine “may promote effective growth and development in a dynamic clinical, training, and research environment” [19]. Medical schools around the world are striving to achieve and demonstrate a greater impact on the health needs of the populations and societies they serve as part of their social accountability strategies [20-23].

For medical research charities, an important rationale for more outcome-based evaluation of the research they fund is to fulfil more effectively the wishes of their benefactors, *e.g.* “the improvement of the physical conditions of mankind” in the case of the Sir Henry Wellcome Trust [24]. For some collection-based charities, such as the UK’s Arthritis Research Campaign, the virtue of demonstrating the outcomes of research they fund lies in that demonstrable outcomes help them compete for contributions [25]. Overall, medical research charities hope that improved understanding of how research funding impacts on health outcomes will enable them to [26]:

• “show accountability and good research governance to their stakeholders;

• enhance public perception and understanding of biomedical science and the scientific process;

• and allow the development of more effective strategies in research and development to increase the likelihood of ‘successful’ research outcomes.”

For EU and UK government agencies, the agenda behind outcome measures transcends health to encompass innovation, economic growth, and social progress. The Lisbon European Council (2000) set out a strategy for making Europe “the most competitive and dynamic knowledge-based economy in the world, capable of sustainable economic growth with more and better jobs and greater social cohesion” [27]. In conjunction with this strategy, the European Commission has argued for “increased and more effective public expenditure [on R&D and innovation]” [28], and for “the development and piloting of indicators designed to measure the social and economic impact of research in general, and of European/ international collaborative research in particular” [29]. In the UK, the research strategy for the National Health Service (NHS) “Best Research for Best Health” aims both “to improve the nation’s health and increase the nation’s wealth” [30], while the performance monitoring framework developed by the UK’s National Institute for Health Research (NIHR) links early performance indicators with longer-term research impacts [31].

In 1989, the UK was the first country in the world to implement a performance-based research funding system, the Research Assessment Exercise (RAE), and since then at least thirteen more countries, including Australia, New Zealand, Hong Kong (China), and several EU countries, have introduced such systems [32]. The RAE was conducted approximately every five years to assess higher education-based research activity for the purpose of allocating core public research funds to universities and other HEIs. Those who performed well had their public research funding increased, while those who underperformed had it decreased. Traditionally, the focus of the RAE has been on “quality”, seen as academic or scholarly excellence and assessed through the criteria of rigour, originality and significance, coupled with other indicators, such as academic esteem, viability of research environments, and research capacity [33]. These criteria have been interpreted in each subject-group (known as “unit of assessment”) by dedicated sub-panels of assessors, who peer-reviewed the different parts of institutional submissions and used a common grading scale to arrive at quantified quality profiles.

In 2014, the RAE will be replaced by a new performance-based research funding system – the Research Excellence Framework (REF). The aims of the REF are similar to the RAE, i.e. primarily to provide a basis for resource allocation, accountability for public investment in research, and benchmarking information and reputational yardsticks for the higher education sector [34]. A major new development in the REF was the decision to base the future allocation of public research funding to universities and other HEIs on, *inter alia*, the social and economic impact of research [35]. The introduction of impact assessment was wrought with controversy as it was perceived by many, especially in social sciences and humanities, as “a threat to researchers’ autonomy and to fundamental academic freedoms” [36]. Given that universities in the UK are independent from the government and that academic professions are self-regulated, elected politicians in the government cannot hold universities directly accountable for the type of research they choose to conduct. Therefore, in order to achieve its goal of increasing the economic and social impact of publicly-funded research within specified time horizons, the government introduced funding incentives for universities to engage in high-impact research. This decision was viewed by many as the government’s attempt to limit universities’ freedom to pursue all forms of research, including research that may not necessarily lead to high impact, and to hold independent academic professions accountable for something that they had not chosen themselves [36].

In academic clinical medicine, the introduction of impact assessment was perceived as less controversial. Due to their focus on translational research and health outcomes, academic physicians and scientists were used to outcome-based indicators, and there had been growing global consensus among medical schools about the need to strengthen their social accountability [22]. The latter is defined by the World Health Organization as "the obligation to direct their [medical schools’] education, research and service activities towards addressing the priority health concerns of the community, region, and/or nation they have the mandate to serve. The priority health concerns are to be identified jointly by governments, health care organisations, health professionals and the public” [20]. Importantly, the notion of social accountability implies that it is measured and reported back to society [23].

The proposals to include indicators of social and economic impact in the UK’s performance-based research funding system were first tested in a pilot exercise run by the Higher Education Funding Council for England (HEFCE) on behalf of four UK higher education funding bodies in 2010. The impact pilot exercise aimed to develop a coherent approach to assessing impact in the REF. It invited participating institutions to submit details of economic and societal impacts in several disciplines (including clinical medicine), in the form of a) an impact statement, and b) case studies of specific impacts, produced using a generic impact template. The impact statement was designed to provide “evidence of the breadth of the unit’s contributions to society or the economy” [37]. It had to include any appropriate indicators of impact, collaboration, and other interactions with research users (such as the NHS, UK and EU government, home and overseas industry, charities, and regional development agencies), which were deemed applicable to the whole subject-group (“unit of assessment”). HEFCE intended to use this information together with research income from key research users to explore the feasibility of developing standardised impact indicators. The case studies were designed to illustrate the unit’s contributions in more detail. Although participating institutions could choose which impacts to include, they were encouraged to submit case studies illustrating a wide range of impacts. For every ten faculty members, participating institutions were asked to submit one case study. The generic impact template for each case study included information on 1) the nature and extent of a specific impact, including appropriate indicators; 2) how the unit’s research activity contributed to this impact; and 3) references to external sources that could corroborate the information about the impact and its underpinning research [37].

For the purpose of the 2010 pilot exercise, HEFCE defined research impact as “any identifiable benefit to, or positive influence on, the economy, society, public policy or services, culture, the environment or quality of life,” and provided HEIs with a “common menu” of impact indicators in the following broad categories [37]:

• Delivering highly skilled people;

• Creating new businesses, improving the performance of existing businesses, or commercialising new products or processes;

• Attracting R&D investment from global business;

• Better informed public policy-making or improved public services;

• Improved patient care or health outcomes;

• Progress towards sustainable development, including environmental sustainability;

• Cultural enrichment, including improved public engagement with science and research;

• Improved social welfare, social cohesion or national security;

• Other quality of life benefits.

On the basis of the evidence gathered through the 2010 pilot exercise [38] and a public consultation [39], the funding bodies decided to invite HEIs to submit, in the next round of research assessment under the REF in 2014, a statement about their approach to, and strategy for, enabling research impact, together with a set of impact case studies containing “a narrative that includes indicators and evidence as appropriate to the case being made” [35]. The definition of impact was more clearly focused on non-academic effects, changes or benefits [34]. It was also decided that the assessment of impact will account for 20% of the overall assessment outcomes, alongside the quality of research outputs (65%) and the vitality of the research environment (15%) [35]. The funding bodies set out the generic assessment criteria of “reach” (or breadth) and “significance” (or intensity) of impact, while the discipline-specific interpretations of these criteria were left to the panels and sub-panels responsible for the actual assessment [40]. The “common menu” of indicators was dropped from the generic guidance, but re-worked forms of it were maintained in the statements of criteria and working methods of the four main panels of assessors, including Panel A, of which clinical medicine is a part [40].

As indicated by the rich body of research on the influence on HEIs of the previous rounds of the RAE, the newly-introduced impact assessment for resource allocation purposes via the REF is likely to affect strategic decisions in HEIs at institutional and departmental level, as well as the individual behaviour of researchers and research teams [41-45]. Given the aims of the REF and the weighting of 20% given to impact in the overall outcome, HEIs in the UK are already keenly aware of the higher stakes involved. Particularly challenging is the fact that the development of impact assessment methodologies has still to address major difficulties, such as that of causality, of operationalisation, of attribution, of tracking long-term impacts, of combining quantitative and qualitative indicators, or of balancing the concern for sensitivity to field and context specificities with that for comparability across cases [12,36,46-51]. In addition, prior to the REF, institutions and individual researchers had not been expected to closely monitor and account for impact-related activities; thus, at the start of the current REF assessment period, impact records, if any, were at best patchy, and fit-for-purpose information and management systems were not available. Some competitively allocated project funding streams (such as those by the Research Councils UK, including the Medical Research Council) introduced explicit requirements to report on engagement, dissemination or knowledge exchange activities. However, as the overlap between these activities and the REF definition of impact was only partial, the systems that had been put in place for this purpose sometimes added to the confusion, rather than making it easier, about reporting on impact for REF purposes. As such, the forthcoming REF is facing serious difficulties in simultaneously attempting to conceptualise, operationalise, and assess impact.

In this article, we critically review and appraise the impact indicators used by HEFCE to inform the 2010 impact pilot exercise, using insights from the relevant literature and empirical data gathered for the University of Oxford’s submission to the pilot exercise in clinical medicine. We show that, while the majority of the proposed indicators have some validity, there are significant challenges in operationalising and measuring these indicators reliably, as well as in comparing evidence of research impact across different cases in a standardised and consistent manner. We argue that this unevenness needs to be addressed early on in the design of methodologies aimed at capturing and assessing impact, and that more work, involving collaboration among research, beneficiary, and funding partners, is required to ensure a research-informed approach to impact assessment in the UK REF and beyond.

In what follows, we begin with a methods section explaining why clinical medicine at the University of Oxford provides a critical opportunity to study impact assessment, and describing the design of the 2010 impact pilot exercise. We then present the empirical data and discuss the strengths and limitations of the proposed impact indicators using insights from the relevant biomedical and research policy literature. Finally, we draw conclusions and make recommendations for the development and validation of impact indicators.

## Methods

**Figure 1:**
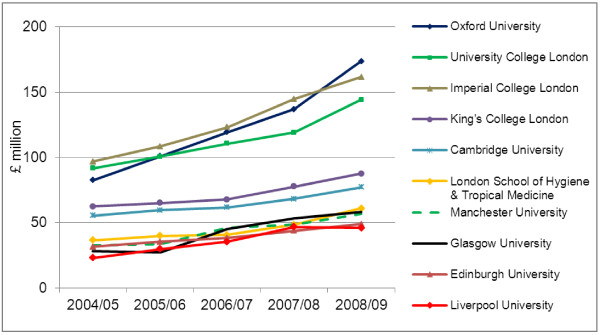
**Top 10 UK HEIs by income from research grants and contracts in clinical medicine, 2004/05-2008/09. **Source: Higher Education Information Database for Institutions (heidi), 2010.

A total of 29 HEIs, including the University of Oxford, participated in the 2010 impact pilot exercise in one or more subject-groups (“units of assessment”): clinical medicine, physics, earth systems and environmental sciences, social work and social policy & administration, and English language & literature [37]. Clinical medicine is the largest subject-group, by research income, in the UK, and as such it provides a critical opportunity for the development and validation of impact indicators. Out of £4,145 million worth of research grants and contracts awarded to UK HEIs in 2008/09 (i.e. at the end of the census period for the pilot exercise), clinical medicine awards accounted for £1,347 million across all HEIs, including £174 million awarded to the University of Oxford alone (Figure 1) [52].

This article uses administrative data from the University of Oxford to operationalise the “common menu” of impact indicators proposed by HEFCE and to test their relevance to research practice in clinical medicine. In 2010, in preparation for the pilot exercise, the university’s Medical Sciences Division collected a first round of relevant administrative data from university sources. In addition, the Division developed an online administrative survey, based on the HEFCE “common menu” of impact indicators; the survey was conducted among the faculty members forming the university’s clinical medicine “unit of assessment”. In 2011, a second round of data was gathered by the authors from the relevant administrative units of the university and publicly available sources specifically for the purpose of the current study including updated data on commercialisation activities, clinical trials, and financial indicators. The university’s Clinical Trials and Research Governance Team reviewed the study and deemed necessary no further ethics committee clearance. Institutional approval for the use of the administrative data used in the paper was secured from the Medical Sciences Division.

In March 2010, instructions and an electronic link to the impact assessment survey were e-mailed to the 289 faculty members who were most likely to participate in the 2014 REF, i.e. those who participated in the 2008 RAE in the “units of assessment” relevant to clinical medicine and who at the moment of the survey had active university e-mail accounts. The survey included 15 open-ended questions structured around the proposed impact indicators, *e.g.* “If in the period between January 2005 and December 2009, you participated on public policy/advisory committees, please indicate how many times and specify their topics and your capacity”. The survey also included a personal details section and three questions prompting respondents to provide open-ended comments or thoughts on the impact indicators, on the impact pilot exercise, and on how the Medical Sciences Division should organise the collection of impact data on a regular basis in the least burdensome way for faculty.

The survey received a total of 139 responses, including four responses after the closing date, which all were accepted for analysis here (48.1% response rate). The achieved response rate was relatively good for voluntary online surveys involving physicians both in the current research setting [53] and elsewhere [54]. Sample proportions by gender, career stage, and staff category were similar to the population surveyed; however, women, early-career researchers, and mid-career researchers were slightly overrepresented, whilst senior researchers were slightly underrepresented; there were also slight discrepancies within staff categories (Table 1). Staff grades from the university’s payroll were used to determine career stage and staff category, and these were unknown for 5 researchers (1.7%), who were no longer on the payroll. Overall, the survey provided a unique and adequate insight into individual and aggregated evidence of impacts and into clinical medicine faculty members’ views on the nature and appropriateness of the impact indicators proposed by HEFCE.

**Table 1:** Demographic and professional characteristics of clinical medicine faculty, 2010

**Characteristic**	**Population (N=289)**	**Sample (n=139)**
**Gender**		
Female	21.5%	22.3%
Male	78.5%	77.7%
**Career stage**		
Senior (professorial)	43.3%	41.0%
Mid-career	43.9%	46.0%
Early-career	11.1%	12.9%
Unknown	1.7%	0.0%
**Staff category**		
Category A, university scientists	71.3%	75.5%
Category A, university clinical academics	18.7%	18.0%
Category C, MRC scientists and NHS clinicians	8.3%	6.5%
Unknown	1.7%	0.0%

## Results and discussion

In the main part of this paper, we assess the scope and relevance to clinical medicine research of a range of measures that operationalise the impact indicators proposed. We unpack each of the potential indicators listed above, track its rise as an aspect of research deemed important by a range of interested parties, discuss some of the measures most commonly used to assess it, and illustrate the strengths and limitations of these measures using data from clinical medicine in Oxford. Data are presented in anonymised and aggregated format and relate to the five-year census period January 2005 – December 2009, unless otherwise stated.

### Delivering highly skilled people

#### Staff movement between academia and industry

Increasing human mobility between academia and industry is a desired goal not only in the UK, but also in EU and OECD countries – it is perceived to enhance knowledge transfer in both directions and to offer better employability and career prospects for researchers [55-57]. The Lambert Review of business-university collaboration in the UK concluded that “the best forms of knowledge transfer involve human interaction” [58]. Moreover, research evidence from the US suggests that inter-sectoral collaborations and staff movements from academic to industrial jobs and *vice versa* are positively associated with researchers’ productivity, most likely, because of the accumulation of scientific and technical human capital in multiple settings [59,60]. There is also some normative guidance on the desirable rate of staff movement. Following the 2005 summit of EU leaders in Hampton Court, the Aho Report on creating an innovative Europe suggested that “[t]en per cent of the workforce in each year should be moving” [61].

Although our analysis of administrative data from Oxford showed minimal full-time movement of tenured researchers between the sectors, a significant number of university researchers worked in industry part-time, on temporary assignments, or in their own time; conversely, many industry researchers held visiting positions in the university. More than one-third of respondents to the impact assessment survey (37%) reported spending time working in, or providing advice and consultancy to, industry, through *ad hoc* research projects and collaborations, long-term industrial-academic partnerships, or permanent appointments in companies’ scientific and advisory committees and non-executive boards. The range of companies included global biotechnology and pharmaceutical companies, small and medium enterprises, and university spin-outs. The amount of time spent in industry by each respondent ranged from several working days or weekends to two months *per annum*.

These data suggest that there is considerable industry demand for the expertise of university researchers, but this demand is not (yet) accompanied by the full-time job changes characteristic of the US-style “revolving door” model of academia-industry employment [62]. Similarly, according to the 2011 Careers in Research Online Survey (CROS) of research staff in UK HEIs, the proportion of staff reporting industry and business placements and secondments across the HE sector is still low (5% and 7%, respectively), while other types of collaborations with industry are more prevalent (36% of the total respondents) [63]. Consequently, in documenting and assessing this area of impact, the absolute number and proportion of academic and research staff who worked part-time in, or provided advice and consultancy to, industry, their roles, and the time spent working in or with industry need to be taken into account.

#### Employment of post-doctoral researchers in industry

The Roberts Review of the supply of people with science, technology, engineering and mathematics skills in the UK acknowledged that post-doctoral and other contract research staff (CRS) offer universities project-based skills, staffing flexibility, new innovative approaches, and other advantages [64]. Nonetheless, limited permanent academic employment opportunities, not only in the UK, but also internationally, cause concerns that CRS may spend a long time moving from one temporary position to another, to the detriment of their professional development, career prospects, and quality of work and life conditions [65]. For example, 14% of the research staff surveyed in CROS 2011 reported having held five or more different contracts of employment as researchers with their current institutions [63]. A further concern arising from this situation is that highly-skilled graduates would be less interested in pursuing research careers [66-69]. Roberts argued against CRS remaining on a series of short-term contracts for a long period of time, and suggested improving CRS training in the skills required either in an academic or industrial career and that “in time… [the industrial career trajectory] should come to be regarded as the ‘default option’ by CRS” [64]. Therefore, whilst bearing in mind that industry demand for higher degree graduates varies widely across sectors and organisations [62], the employment of post-doctoral researchers in permanent positions in industry may be used as an indicator of the ability of universities to provide CRS with skills and career development opportunities (*e.g.* industrial placements and secondments) to pursue a corporate research career.

In trying to operationalise this indicator in Oxford, we found no clear definition for post-doctoral research positions in the current human resources (HR) practices, which seems to be a common limitation of the higher education HR practices in many countries [68]. For this reason, we examined data declared on the leaver’s form to gather information on the destinations of leaving CRS on grades 7 and 8 as a proxy for leaving post-doctoral researchers. During the five-year census period, 1013 such CRS left clinical departments; the known destinations of 762 CRS, who declared going into regular employment or study, are as follows:

• 57% – education and research institutions in the UK and overseas,

• 22% – health services in the UK and overseas,

• 9% – private industry/commerce or self-employed in the UK,

• 12% – other employment in the UK and overseas.

Another important limitation is the absence of any information on whether the subsequent jobs of CRS are permanent or short-term. If a move into industry is accompanied by a series of short-term contracts, then this could be interpreted as a failure of the university in question to help CRS develop skills for a more permanent career; *vice versa*, a move to another education or research institution on a permanent contract could be interpreted as a success. Given the lack of reliable data, developing a clear, applicable measure of the employment of post-doctoral researchers in industry seems problematic.

Concomitantly, these data draw attention to the movement of physician-scientists and other highly-skilled individuals who are trained to perform translational research between universities and health services. Bridging research and clinical practice by increasing the pool of translational investigators is high on the agenda on both sides of the Atlantic [70-72]. In the UK, for example, the NIHR has established Academic Clinical Fellowships, Clinical Lectureships, In Practice Fellowships, and Clinician Scientist Awards to promote integrated academic and clinical careers at the pre-doctoral, doctoral, and post-doctoral levels [73]. The efforts of universities and their partner teaching hospitals to increase the pool of translational investigators can, thus, be assessed by measuring the number of such awards.

### Creating new businesses, improving the performance of existing businesses, or commercialising new products or processes

#### Research contracts and income from industry

Whilst different types of research can have impact on the economy, the Cooksey Review of UK health research funding concluded that more opportunities to create additional health and wealth benefits from research lie in translational and applied research [74]. The review recommended that, while sustaining the current funding levels for basic science, “future increases in funding should be weighted towards translational and applied research until a more balanced portfolio is achieved” [74]. Empirical studies suggest that scientists with industry funding conduct more applied research and, contrary to a popular assumption that industry funding negatively affects traditional scientific outputs such as publications, they also produce more scientific publications than scientists without industry funding [75,76]. Therefore, research contracts and income from industry can be used as an indicator of potential health and wealth benefits for the economy, and an increased level of industry funding may also positively affect the traditional scientific outputs.

According to the Higher Education Statistics Agency (HESA), income from research grants and contracts in clinical medicine from industry in 2008/09 was higher in Oxford (£20.3 million) than in any other UK HEI except Imperial College London (£26.1 million); it accounted for 11.7% and 10.3% of external research income in Oxford and all UK HEIs respectively (Table 2) [52]. While research income from industry is a useful indicator, it does not allow assessment of the potential health and wealth benefits of the overwhelming majority of clinical medicine research in UK HEIs, which is funded by the public either through government agencies or charities. Yet, the pharmaceutical industry is highly dependent on publicly-funded research, in particular basic research [77]. US studies found that 31% of drugs and medical products could not have been developed (without substantial delay) in the absence of recent academic research [78], and that 79.1% of the papers cited by US industry drug and medicine patents were outputs of publicly-funded academic science [79]. Most citations in patents are journal references and may be used to develop robust bibliometric indicators [80]. Similar to the use of bibliometric analyses of highly-cited publications by the NIHR to support the procurement of Biomedical Research Centres in England [81], HEFCE and other research funders can use bibliometric analysis of citations in patents to assess the contribution of publicly-funded clinical medicine research to innovations in industry.

**Table 2:** Income from research grants and contracts in clinical medicine by sponsor type, 2008/09

**Sponsor type**	**University of Oxford**		**All UK HEIs**	
	**£M**	**%**	**£M**	**%**
**Government**	**53.3**	**30.7%**	**558.9**	**41.5%**
UK Research Councils, Royal Society & British Academy	25.4	14.6%	222.5	16.5%
UK central government bodies/ local authorities, health & hospital authorities	19.7	11.3%	282.0	20.9%
EU government bodies	8.2	4.8%	54.4	4.0%
**Charity**	**89.0**	**51.2%**	**595.4**	**44.2%**
UK-based charities (open competitive process)	77.6	44.7%	454.7	33.8%
UK-based charities (other)	2.4	1.4%	78.3	5.8%
EU-based charities (open competitive process)	0.2	0.1%	3.0	0.2%
Non-EU-based charities (open competitive process)	8.7	5.0%	59.4	4.4%
**Industry**	**20.3**	**11.7%**	**138.1**	**10.3%**
UK industry, commerce & public corporations	4.3	2.5%	84.0	6.2%
EU industry, commerce & public corporations	1.2	0.7%	14.8	1.1%
Non-EU industry, commerce & public corporations	14.8	8.5%	39.4	2.9%
**Other**	**11.0**	**6.3%**	**54.4**	**4.0%**
EU other	1.4	0.8%	5.9	0.4%
Non-EU other	9.6	5.5%	38.6	2.9%
Other sources	0.0	0.0%	9.9	0.7%
**Total**	**173.6**	**100.0%**	**1,346.9**	**100.0%**

Source: Higher Education Information Database for Institutions (heidi), 2010.

#### Collaborative research with industry measured through co-authored outputs

Measuring co-authored publications is a well-grounded, although not comprehensive, way of assessing collaborative research with industry. There is evidence from the US that university-industry collaborations tend to be driven by industry’s agendas and that the resulting co-authored publications are less basic and more applied than universities would produce otherwise [82]. Moreover, empirical studies from different countries suggest that co-authored publications are positively associated with researchers’ productivity in terms of the number of publications and citations, but there is no conclusive evidence on whether co-authored publications are more likely to appear in journals with higher or lower impact factors than university-only papers [82-84]. However, there is evidence to the effect that measuring co-authored publications is not a comprehensive way of assessing university-industry collaboration. For example, in the case of a Swedish medical university both industry funding and co-authorship indicators were shown to provide incomplete results [85], and a study of multi-disciplinary research teams in Germany concluded that co-authorships could account for about half of the actual collaborations [86]. On the whole, analysing co-authored publications can be a useful, but by no means exhaustive, way of assessing university-industry collaboration.

In Oxford, 30% of respondents to the impact assessment survey reported co-authoring one or more publications with colleagues from industry over the five-year census period (averaging 3.5 publications per respondent). A number of respondents also mentioned industry-funded publications with only academic authors, and further publications acknowledging industry funding. Furthermore, our analysis of administrative records revealed collaborative relationships with industry that were not necessarily accompanied by industry funding or co-authored publications, but could be accounted for by less conventional indicators such as memoranda of understanding, confidentiality agreements, collaboration agreements, data/material transfer agreements, and visitor agreements. Hence, it can be suggested that impact narratives should include additional indicators, such as those mentioned above, in order to capture a fuller range of collaborative relationships with industry.

#### Income from intellectual property

The large-scale exploitation of intellectual property by universities is relatively new to the UK. The right of first refusal on the commercialisation of publicly-funded research, held for many decades by the British Technology Group, was only rescinded in 1985. As a consequence, universities gained direct control over, and exploitation of, their intellectual property, with a view to generating income for themselves and contributing to national wealth creation [87]. Not with standing conflicting views on whether commercialisation can harm or promote basic research and education [88], encouraging and providing universities with funding to engage more actively in the exploitation of intellectual property has consistently been the government’s policy [58,89-91]. A 2009 HEFCE report concluded that there was strong support for knowledge exchange in HEIs and that it was increasingly seen by HEIs as complementary to their traditional research and education activities [92]. However, the report stated that revenues from intellectual property represented a very small proportion of income from knowledge exchange [92]. Even in the US, with its long history of university technology transfer, revenues from intellectual property are rather small compared to research expenditure – most universities break even or make only a small amount from their investment in technology transfer activities [93]. Moreover, the total revenue from technology transfer in US universities and research hospitals is “dominated by a few very large royalties from fewer than 1% of total patents” and is uneven over time: “pharmaceutical royalties are high – but very rare,” and “equity cash-ins from spin-outs are only occasionally large, and are one-time” [93].

Oxford academics were already entrepreneurial in the 1950s, but it was not until the establishment of the university’s technology transfer office in 1988, Isis Innovation, that Oxford became “arguably the UK’s most entrepreneurial university” [94]. Isis Innovation helps academics and researchers commercialise their work through patenting and licensing, material sales, spin-out companies, and consulting. Between 2004/05 and 2008/09, the total project income of Isis Innovation was £9.8 million, 50% of which may be attributed to clinical medicine. Precise attribution is somewhat problematic, because many deals were multidisciplinary (involving clinical, pre-clinical, and bioengineering departments) and the current information system does not allow accurate dissection of the existing data in relation to academic departments. Given that in this five-year period Oxford’s clinical medicine research expenditure amounted to £612.9 million [52], intellectual property return on research expenditure was approximately 0.8%. Overall, the total income from intellectual property is a useful indicator, but currently it is problematic to attribute it accurately between multiple departments and it represents only a small percentage compared to research expenditure. It seems more expedient to assess it on the level of the HEI as a whole and in individual subject-groups use case studies only of the most successful commercialisation activities.

#### Success measures for spin-out companies

Technology transfer through spin-out companies has advantages over licensing when the nature of new technology may not be easily patented and when universities seek a greater return on their intellectual property in the long run [95]. For all that, spinning out university companies is rather resource-intensive, in terms of both funding and the inventor’s time. The Lambert Review pointed out that, due to the ready availability of funding for high-tech start-ups and an undue emphasis on spin-outs as a source of employment, too many university spin-outs were being created in the UK compared to North America, including some of low quality [58]. Lambert suggested shifting the balance of commercialisation activities towards licensing and concentrating on high-quality spin-outs, as measured by their ability to attract external private equity [58]. In light of this, together with growth in revenue or numbers of employees, as proposed by HEFCE, external private equity backing can be taken as an important success measure for spin-out companies.

In Oxford, Isis Innovation spins out more biomedical companies and attracts more investment than any other UK university [96]. In doing so, it tries to replicate the success of PowderJect Pharmaceuticals – a vaccine, drug and diagnostics delivery company spun out from the university in 1993 and acquired ten years later by Chiron Corporation for $800 million [97]. During the census period, Isis Innovation supported the creation of 25 spin-out companies, of which 8 related to clinical medicine in the following areas [98]:

• Celleron – developing targeted cancer medicines;

• TΔS – developing ketone bodies as medical foods to increase physical and mental performance;

• Particle Therapeutics– needleless delivery of therapeutic molecules across the skin;

• Cytox– pre-symptomatic diagnosis of Alzheimer's Disease;

• Clinox– provider of phase I/II trials in oncology;

• Oxford BioDynamics– detection of aberrant gene expression (prognosis and diagnosis);

• Oxford-Emergent TB Consortium –joint venture with Emergent BioSolutions to develop tuberculosis vaccine candidate, MVA85A;

• Organox – portable device to preserve livers for transplantation for up to 3 days.

During the census period, these spin-out companies employed a small number of people (on average 18 people *per annum*) and generated no income from sales. Nevertheless, they raised a total of £15.7 million in external private equity, indicating investor confidence in future revenue growth. They also spent a significant proportion of their expenditure on R&D in universities or technology consultancy companies, contributing to job creation in these sectors. As one respondent to the impact assessment survey argued, the success of spin-out companies is not measurable over a five-year period: “our 2 spin out companies took, in one case 7 years, and in the other more than 10 years, before they [could] realise their full value by either bringing products, such as new drugs, to market or by being successfully sold or by going public”. It can, thus, be argued that the success of spin-out companies should be evaluated over a longer period of time and that quality measures, such as the ability to attract external private investment, should also be taken into account.

#### Patents granted/licences awarded and brought to market

Patenting and patent licensing is not as resource-intensive as spinning out companies, but seeking to increase the levels of patenting and licensing may lead to unintended consequences. For example, Henderson *et al.* argued that the Bayh-Dole Act, which incentivised US universities to patent, resulted in the decline of the quality of university patents, as measured by citations in subsequent patents [99,100]. Others demonstrated that increasing the number of inventions, for which patent applications were made and licences sought, reduced the average “yield” of these commercialisation activities [101]. Also, there are concerns that excessive proliferation of intellectual property rights may deter innovation [102], and that misconceptions in the value of intellectual property may inhibit collaboration with industry [103]. Finally, beyond a certain level, higher levels of patenting are negatively associated with academic productivity in terms of publications [104] and their “basicness” [105]. For these reasons, patent numbers may be noisy indicators of knowledge transfer and commercialisation. In order to minimise the negative unintended consequences of excessive patenting, it is important to take into account the quality of patents, which can be measured by licences or by citations in subsequent patents. Licences are an appealing measure of quality because they indicate the economic value of patents and they are enforced by the competing interests of licensors and licensees [106]. Citations in subsequent patents are indicative of the technological importance of the antecedent patent, i.e. they draw on the knowledge embodied in the antecedent patent, and/or they may indicate that the antecedent patent had opened up a new field of inventive activity [100].

During the financial years 2004/05 to 2008/09, Isis Innovation filed a total of 293 new patent applications from university researchers in Oxford, of which 63 were based on disclosures from clinical medicine departments. Within the same period 276 licence deals were completed, including 61 that were related to projects derived from clinical medicine disclosures. There are no comparative data for patents and licences in clinical medicine, but in life sciences in general Oxford publishes more patents than any other university in the UK [96]; and overall Isis Innovation files more international patent applications to the World Intellectual Property Organization under the Patent Cooperation Treaty than any other university in Europe [107]. Given that there is a significant variability between UK universities in research power and commercialisation activity, it is useful to take into account universities’ research power when comparing the absolute number of patents or licences [96]. Moreover, given that the absolute number of patents does not provide information about their quality, it would be more effective using this measure in combination with additional measures, such as income from licencing and citations in subsequent patents.

### Attracting R&D investment from global business

#### Research income from overseas business

The globalisation of R&D has grown substantially over the past decades [108] to the effect that increasingly R&D is being outsourced to Asia, particularly China and India. This promotes technological change and diffusion of technological advances worldwide, but raises concerns about the future of the domestic knowledge base in OECD countries and the competitiveness of their economies [109]. In addressing such concerns, the UK’s national health research strategy, Best Research for Best Health, aims “to make the UK the best place in the world for health research, development and innovation” [30]. Empirical research has found a positive association between the productivity of academic research in host countries (as measured by the number of publications) and the level of foreign R&D investment, and that academically stronger countries attract companies with a stronger science orientation in their R&D activities [110]. It follows that research income from overseas companies may be indicative of the contribution of a university’s research to both the competitiveness of the domestic economy and global economic growth.

**Figure 2:**
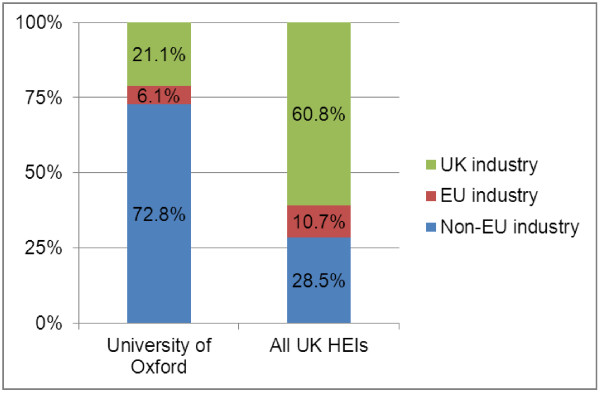
**Income from research grants and contracts in clinical medicine from industry, 2008/09. **Source: Higher Education Information Database for Institutions (heidi), 2010.

In 2008/09, UK HEIs attracted £54.2 million of research income from EU and other overseas industry, of which Oxford accounted for £16 million (Table 2). While the percentage of non-EU overseas industry funding in Oxford is more than twice as high as in all UK HEIs combined, the percentage of EU industry funding in Oxford is lower (Figure 2). Overall, Oxford attracts significantly more R&D investment from global business than the average UK HEI, as 78.9% of Oxford’s industry research income comes from overseas industry.

### Better-informed public policy-making or improved public services

#### Changes to legislation/regulations/government policy

It has been argued that “the use of research knowledge to inform decision-making, not a change in health status, constitutes the most important generic measure of the impact of research that can be assessed routinely” [111]. Yet, the attribution of policy changes to particular research projects or funding is a methodologically-challenging and research-intensive task [112]. A recent review of research impact on policy concluded that “the interests of various stakeholders such as politicians, public servants, religious groups, pharmaceutical and diagnostic companies, and health professionals may often run counter to the introduction of new research findings, thus, affecting policy making, budgeting, and implementation” [113]. Moreover, given the collective nature of decision-making in democratic societies, it is challenging to attribute changes in legislation, regulation, or government policy to certain teams or individuals. As Weiss argued as early as 1979, “it probably takes an extraordinary concatenation of circumstances for research to influence policy directly” [114]. It follows that, for the most part, the assessment of research impact on policy requires the triangulation of data from a number of stakeholders using qualitative methods and a case study approach [113,115].

In Oxford, 9% of respondents to the impact assessment survey reported influencing changes to legislation, regulations, or government policy on the national, European, or international levels. The range of their activities mainly concerned the provision of thought leadership and advice to various governmental, professional, and advisory bodies. For example, one respondent mentioned a secondment to the Department of Health as a national clinical director to write and implement government policy for a major clinical service. In another example, a respondent mentioned direct contacts with the World Health Organization, in his/her role as president of an international professional society, leading to updates to guidelines for a complex area of practice and research. Other respondents also mentioned instances of their papers being used as evidence during public policy deliberations, or themselves participating in policy advocacy campaigns. It is important to note that several respondents were sceptical about the feasibility of attributing policy changes to one’s research-based activity, and one respondent admitted to not knowing how to prove that his/her policy advocacy had contributed to policy change. A further limitation arises from the fact that assessing only documented influence on changes to legislation and regulation (*e.g.*, on the basis of references made to research outputs in policy documents) will overlook the more complex, qualitative aspects of academics’ participation in the democratic deliberation and implementation of public policies. Taking all these into account, it seems to be more inclusive and less burdensome to assess researchers’ participation in public policy processes, for example, through provision of advice and leadership, rather than attempting to separate out the contribution of specific research in achieving changes to legislation and regulation.

#### Participation on public policy advisory committees

Universities can play an important role in ensuring that government policies “are forward-looking and shaped by the evidence rather than a response to short-term pressures” [116]. University academics generate an evidence base for public policy [117] and can facilitate the passage of new research knowledge into the evidence base of concrete policies. Their participation on public policy/advisory committees may smooth that transition and, thus, can be regarded an indicator of impact. Taking into account the multi-stakeholder nature of public policy-making in democratic societies, this indicator includes not only public policy/advisory committees of government agencies, but also those of supranational organisations, professional bodies, charities, and other stakeholders involved in public policy-making.

In Oxford, 42% of respondents to the impact assessment survey reported participating on public policy/advisory committees of various UK, overseas, and supranational organisations, including the World Health Organization, the European Space Agency, UK Department of Health and other government agencies, UK parliamentary committees, political parties, professional associations, medical charities, universities, and schools. The significance of these roles ranged from elected or appointed senior leadership positions to ordinary committee membership or *ad hoc* advisory roles. Importantly, several respondents suggested that participation on the advisory committees of charities should be included in impact assessment, although they had reservations about the additional burden of work that keeping full, accurate records of all these activities would require. One respondent noted:

“I sit as a trustee of probably up to 12 charities, most of which have something to do with medical research. I think that sort of contribution is at least as important as contributions made to government activities. [However] I think it would be intolerable to have to keep a detailed account of all such activities and how would they be ranked relative to each other.”

#### Influence on public policy debate

UK scientists see the news media as having a significant impact on public debate and many regard speaking to the media as the most effective method of communicating with the public and influencing policy-makers [118]. Moreover, a survey of interactions with the mass media among researchers in France, Germany, Japan, the UK, and the US concluded that “the scientists most involved in these interactions tend to be scientifically productive, have leadership roles, and… that they perceive the interactions to have more positive than negative outcomes” [119]. Nonetheless, there may be a bias in reporting on the side of both the mass media and research institutions. Media interest in research may be linked to profound concerns about the evidence base for public policy, or with genuine interest in new knowledge, but it also feeds on what a respondent to a recent study called the “scientific entertainment value” of research [112]. A UK study suggested that “newspapers underreported randomised trials, emphasised bad news from observational studies, and ignored research from developing countries” [120]. A US study showed that news stories about new medical interventions are often inaccurate, imbalanced, or incomplete, and that as a result “may have a profound—and perhaps harmful— impact on health care consumers” [121]. But it is not only journalists who may be over-enthusiastic about weak science: press-releases by US academic medical centres themselves may have “a tendency to overstate the importance and downplay (or ignore) the limitations of research” [122]. For this reason, any media impact indicators should take into account not only the number of citations and appearances, but also their quality, and should be used with caution.

In Oxford, 42% of respondents to the impact assessment survey reported appearing in the local, national, or international mass media during the five-year census period. Although no direct comparison is possible, this may be lower than implied by an earlier survey of epidemiology and stem cell researchers in the top five R&D countries for these fields, of whom 69% had been involved in interactions with the mass media in the last three years [119]. The range and magnitude of media impact in the context of this study can be assessed using data from the university’s Press & Information Office. For example, Table 3 shows the coverage of scientists from clinical medicine departments in conventional media in 2009. In addition, since October 2008, university clinical scientists have been able to disseminate their research through the iTunes U website, which in 2009 carried 6 free-to-download podcasts relevant to clinical medicine: Cancer, Cancer in the Developing World, Childhood Diseases, Clinical Trials in Resource-Limited Settings, Pharmaceutical Industry, and Vaccine Research [123]. The number of downloads for such podcasts can be obtained from Apple Inc. and compared with other universities.

**Table 3:** Media coverage of scientists from clinical medicine departments, 2009

**Type of media**	**Stories**	
	**n**	**%**
UK & international press	390	62.3%
UK & international online news*	123	19.6%
UK radio	60	9.6%
UK television	53	8.5%
**Total**	**626**	**100.0%**

*Unknown whether a story appeared in hard copy as well.

Source: Press & Information Office, University of Oxford.

At the same time, it is important to note that media coverage does not necessarily lead to influence on public policy debate. Only 12% of respondents to the impact assessment survey reported perceptions of influencing public policy debate. Similar to some of the previous indicators, influence on public policy is a subjective indicator, and media coverage is only a weak measure of it. An important aspect of influencing public debates cannot be captured by such indicators: the potentially transformative influence of research on public discourses about policy and clinical practice includes shaping the ways in which such discourses frame problems and their solutions, and slowly changing the language in which they describe the social and physical worlds.

### Improved patient care or health outcomes

#### Research income from the NHS and medical research charities

Competitively-awarded NHS and charity funding is predicated on the high quality of research and benefits for patients. The NHS Constitution puts research at its core because “[r]esearch enables the NHS to improve the current and future health of the people it serves” [124]. Likewise, medical research charities aim to fund research that is important for the advancement of medical care [125]. Until the creation of the NIHR in 2006, NHS R&D funding was allocated to sustain the level of activity in large teaching hospitals and it is argued to have been “far more a product of history, politics and pragmatic judgments than any rational analysis” [126]. Following the creation of the NIHR, the majority of NHS R&D funding is now awarded competitively to NHS/university partnerships on the basis of peer review and bibliometric indicators [81]. Medical research charities have long been using peer review and research output indicators to allocate their research funding. In the run-up to the previous Research Assessment Exercises they urged government, the NHS and universities “to favour peer-reviewed charity research above funding that is not awarded competitively” [125]. Crucially, medical research charities aim to allocate research funding in order to achieve the greatest impact, and in fact they championed the idea of assessing the long-term benefits from health research in the UK [25]. Yet, there is evidence that competitively-awarded NHS or charity research funding does not necessarily achieve impact. An extensive assessment of the impact of the NHS Health Technology Assessment Programme found that some of its projects achieved virtually no societal or economic impact [127]. It remains to be seen what proportion of competitively-awarded research funding predicated on impact actually achieves impact and which NHS bodies or medical research charities are most successful in funding research that achieves greater impact.

Although the validity and reliability of using competitively-awarded research funding as a proxy indicator of impact requires further investigation, our study suggests that it is feasible to collect the required data and that this can be done with minimal burden on academic staff. HEFCE has recently recognised the competitive nature of NHS research funding, and to this effect the Higher Education Statistics Agency (HESA) is tasked with reporting it specifically alongside other competitively-awarded funding [34]. Charity funding is the most important source of external research income in UK HEIs and its overwhelming majority is awarded competitively. In 2008/09, charity funding accounted for 51.2% and 44.2% of clinical medicine external research income in Oxford and all UK HEIs combined respectively (Table 2). Since the introduction of competitive mechanisms to allocate NHS R&D funding, NHS/university partnerships in Oxford have been successful in obtaining major awards, such as a NIHR Comprehensive Biomedical Research Centre and NIHR Biomedical Research Unit. But if we look at the percentage of research income from “UK central government bodies, local authorities, and health and hospital authorities” in Table 2, which mainly reflects research income from the NHS, it is still almost twice as low in Oxford (11.3%) as in all UK HEIs combined (20.9%).

#### Measures of improved health services

In the UK, up to 5%-10% of the medical workforce is made up of clinical academics – university employees who, in addition to their teaching and research duties, have honorary contracts with the NHS as practicing doctors [128]. As of 2009, there were over 125 full time equivalent (FTE) university-employed clinical academics in Oxford, who represented 4% of the clinical academic workforce in the UK [128]. They play a leading role in translating research from bench to bedside and make a significant contribution to the delivery and improvement of health services, especially those that are highly specialised and technology-intensive. Respondents to the impact assessment survey stressed the link between research and their own professional development, enabling state-of-the-art care for patients, which would otherwise take years to be established through changes to clinical guidelines. As we argued elsewhere, a strong link between practice, as *praxis*, and research involves their “organic” synergy in practitioners’ everyday professional lives, “which is more than having effective traffic of information between two otherwise discrete communities and activities” [129].

Importantly, a number of respondents commented on impact assessment as an opportunity for better recognition of the clinical aspects of their work. They argued that the conventional indicators of research output disadvantaged clinical academics, compared to full-time university scientists, who were described as having more time for basic research and better chances to publish it in journals with high impact factors. An anaesthetist, who was dissatisfied that in the previous RAE his/her specialty was judged solely on the basis of research outputs, commented:

“If these [impact] measures are introduced… it will become impossible for either universities locally or HEFCE nationally to ignore the clinical contributions of anaesthetists and focus narrowly upon research outputs.”

Overall, 13% of respondents reported improving health services, mainly in their local area. The range of impacts reported included: improving diagnostics and drug response prediction, fulfilling previously unmet clinical needs, making highly-specialised services more accessible for the local communities, applying new recovery and rehabilitation strategies, and reducing waiting times and treatment costs. Given the wide range of impacts reported, it does not seem feasible to have one standardised measure of improved health services, and so, like in other practice-oriented fields, case studies can be a useful method to capture such impacts [130]. Nonetheless, some standardised measures could be used as a proxy for the impact of universities on the delivery of health services, *e.g.* the number of university-employed clinical academics, their time (programmed activities) devoted to clinical work, and their translational research leadership and esteem as measured by NHS Clinical Excellence Awards and NIHR Senior Investigator Awards.

#### Changes to clinical or healthcare training, practice or guidelines

Even though individual clinicians can adopt research findings directly from academic publications, mass adoption at a national or international level is usually facilitated by clinical guidelines and other evidence-based recommendations. Clinical guidelines, such as those produced by the National Institute for Health and Clinical Excellence (NICE), aim to improve the quality of healthcare in the UK by providing clinical recommendations based on the best available evidence [131]. Consequently, references to research in clinical and practice guidelines can be used as indicators of the likely application or adoption of research findings in clinical practice [132]. Although a citation in clinical guidelines does not guarantee an impact on health, it demonstrates a peer perception of research utility and can be considered as an intermediate outcome [26]. Research by the Wellcome Trust, the Medical Research Council (MRC), and the NIHR demonstrated the feasibility of employing conventional bibliometric analysis of research papers cited on clinical guidelines to assess the impact of biomedical research on healthcare policy and practice [24,26,133].

In Oxford, 20% of respondents to the impact assessment survey reported that their work had resulted in changes to clinical or healthcare training, practice or guidelines at a local, national, European, or international level. These reports were supported with evidence both of output (references in published guidelines) and of process (direct participation in the production of guidelines).The majority of respondents mentioned that their research had been cited by, or that they participated in the working groups or committees of, the NICE, UK Royal Colleges, international professional associations, the World Health Organization, and other relevant bodies. A number of respondents also mentioned that their research was cited in NHS National Service Frameworks and Strategies. Inclusion in use-oriented systematic reviews, such as Cochrane Reviews, which screen studies for quality and relevance, was mentioned as another possible measure of impact. Whilst clinical guidelines, official recommendations, and systematic reviews lend themselves well to bibliometric analysis, some other types of evidence mentioned by respondents (*e.g.* leading on a new MSc course for practising physicians, developing a section of the NHS Evidence web service, or proposing a new classification system for an infectious tropical disease) favour case studies. A case study method may be appropriate for claims about impacts on training and professional standards, accreditation frameworks, curriculum and teaching materials and textbooks, and pedagogical approaches in medical education. For these reasons, a mixed method approach, encompassing both bibliometric analysis and case studies, can be used to assess this area of impact.

#### Development of new or improved drugs, treatments or other medical interventions; numbers of advanced phase clinical trials

The clinical trial in human beings is a valid and apt indicator for the development of medical interventions because currently it is “the preferred method in the evaluation of medical interventions” [134], representing “a key research activity with the potential to improve the quality of health care and control costs through careful comparison of alternative treatments” [135]. Moreover, experimental animal models and other pre-clinical studies can be assessed on the basis of whether they have progressed to clinical trials, and the latter can be assessed on the basis of the benefits associated with each phase of clinical trials. Even though some medical interventions can be difficult to classify, clinical trials are conventionally divided into phases I-IV. Phase I trials are generally designed as pharmacology studies; phase II trials are therapeutic exploratory investigations; phase III trials are assessments of the effectiveness of the new intervention; and phase IV trials are investigations into uncommon adverse effects of the new intervention [134]. Given the strategic intention of the NIHR to increase the number of people in the UK participating in clinical trials and the health benefits of participating [30], the number of participants in clinical trials is also an important indicator for the development of medical interventions.

The University of Oxford runs one of the UK’s largest clinical trials programmes in collaboration with many academic health centres in the UK and overseas. There are eight clinical trials units that have been awarded UK Clinical Research Collaboration (UKCRC) Registration, which recognises their capability to coordinate multi-centre clinical trials to a required standard [136]. During the census period, the university sponsored or participated as a research site in 140 clinical trials involving 154,888 participants (Table 4). According to the impact assessment survey, 32% of respondents reported contributing to the development of new or improved drugs, treatments or other medical interventions. As one respondent noted, however, it is important to give appropriate credit to early-phase translational research and basic science, which underpin advanced phase clinical trials with the most immediate patient benefits:

**Table 4:** University of Oxford sponsored clinical trials active, 2005/09

**Topic area**	**Number of trials**	**Phase**					**Number of participants**
		**I**	**II**	**III**	**IV**	**N/A**	
Cancer	7		1	4	1	1	27314*
Cardiac/circulation	8		1	4	3		56335
Diabetes	7				3	4	16350*
Infectious disease	25	19	3		1	1	1737*
Neo/perinatal	4			1	2	1	7220
Nervous system	2			2			312
Obstetrics/gynaecology	2			1	1		240
Paediatrics	17		8	2	6	1	5510
Public health/primary care	1				1		180
Psychiatry	8	1	2		4	1	1853
Renal	2			1	1		848
Respiratory medicine	4		1	1	2		686
Tropical medicines	53	9	15	8	7	14	36303*
**Total**	**140**	**29**	**31**	**24**	**32**	**23**	**154888**

*incomplete data.

Source: Clinical Trials and Research Governance Team, University of Oxford.

“impact assessment needs to consider carefully the various stages of translational research so as to award credit correctly to those who have devised and brought to clinical evaluation new interventions, rather than giving disproportionate credit to those who undertake late stage evaluation of technologies invented by others.”

#### Changes to public behaviour

There is a growing recognition in medicine and public health that many diseases can be prevented or have their effects lessened by making and maintaining changes to public behaviour, and that demands and responsibilities for such changes should be placed not only on the individual, but also on the health system, the community, and the social and political context [137]. It is a valid expectation on the part of research funders that the medical research community should promote health-enhancing changes to public behaviour. At the same time, the measurement of health behaviours is challenging [137], and so is the attribution of behavioural change to specific research findings [25].

In Oxford, 7% of respondents to the impact assessment survey reported, or expressed hope, that their work had resulted in health-enhancing changes to public behaviour. For example, one respondent assessed that since the implementation of the National Stroke Strategy and the establishment of a new academically-led Stroke Unit in the Oxford John Radcliffe Hospital:

“[stroke] has become an emergency treatment, patients are arriving much more quickly and the public attitude towards stroke has changed insofar as patients now recognize it as a medical emergency.”

Another respondent cited an increased referral rate from UK regions and Ireland to demonstrate a greater awareness of a new life-extending treatment:

“by initiating the FOXFIRE national clinical trial, patients and relatives are seeking the availability of ‘radio-embolisation’ treatment at centres such as Oxford.”

Yet others cited activities, which they hoped would change health-risk behaviours:

• “I would hope that our work on the adverse effects of obesity on heart and arteries has led to increased awareness of obesity as a risk factor”

• “[I was a] Member of Tobacco Control Forum that advised on legislation on smoking ban in restaurants and public houses”

• “I have participated in the Cancer Research UK SunSMART campaign that aims to increase public awareness of exposure to sunlight and the risks of developing skin cancer.”

Given that it is extremely challenging to compare such diverse behavioural changes and verify to what extent they are attributable to specific research, case studies, complemented by standardised measures of health outcomes, can be used to describe such changes, explore their connections with underpinning research, and estimate their potential health benefits. For more robust conclusions, however, more specialised and adequately resourced impact evaluation studies would be required.

#### Measures of improved health outcomes

Research can lead to improved health outcomes through many different pathways, including improvements in health services, changes to clinical guidelines and training, development of new drugs and treatments, and changes to public behaviour as discussed above, but also through the development of new methods to measure health status [6]. Despite the multitude of pathways and disease states, there are standardised measures for assessing health outcomes, such as Quality Adjusted Life Years (QALYs), Disability Adjusted Life Years (DALYs), Health Adjusted Life Expectancy (HALE), and Medical Outcomes Study 36-item Short-Form Health Survey (SF-36) that can be used in research impact assessment [6,18,25]. The key advantage of such measures is that “they serve as a common metric to allow funders to assess the value of an investment across disease states” [18].

In Oxford, 17% of respondents to the impact assessment survey reported that their work had directly or indirectly improved health outcomes in the UK and/or overseas. The range of suggested improvements included reduction in mortality, morbidity, and accumulated disability; improvements in the quality of life of patients and their carers; as well as the development of new disease-specific patient reported outcome measures (PROMs). For all that, none of the respondents were able to provide quantified outcome measures and many expressed concerns that it was difficult to attribute, verify, quantify, and meaningfully compare improvements in health outcomes across various diseases and populations. For example, how does clinical islet transplantation leading to insulin-independency and reversal of life-threatening hypoglycaemic unawareness in UK patients, cited by one respondent, compare to research on the national introduction of the pneumococcal vaccine in the Gambia, cited by another respondent? These responses suggest that there is a need for research funders and the medical research community to develop uniform guidelines for the identification of the populations who can benefit from various interventions, the level of benefit, and the extent of implementation. Provided such guidelines are developed, the standardised measures of health outcomes such as QALYs, DALYs, or HALE can be employed by universities to make initial estimates of health benefits, which may be subsequently validated by research funders and used in conjunction with case studies.

### Cultural enrichment, including improved public engagement with science and research

#### Increased levels of public engagement with science and research

Public engagement, or science communication and public understanding of science, as it is also known, is an inclusive term that refers to “the many ways in which higher education institutions and their staff and students can connect and share their work with the public.” [138]. The Concordat for Engaging the Public with Research, which is signed and supported by major UK funders and stakeholders, recognises “the importance of public engagement to help maximise the social and economic impact of UK research” and suggests that “UK research organisations have a strategic commitment to public engagement” [139]. Likewise, the NIHR has a strategic commitment to involving patients and the public in all stages of NHS R&D, because it sees such engagement as leading “to research that is more relevant to people’s needs and concerns, more reliable and more likely to be put into practice”[30]. For many years, various research funding organisations, including charities, have been running schemes of additional funding for public engagement and outreach projects; the amount and number of such awards may be a proxy for universities’ interest in public engagement activity.

At the same time, a study by the Royal Society found that, in general, scientists did not prioritise public engagement activities because of the need to spend more time on research [140]. They did, however, express willingness to engage more with the public and believed that including public engagement in the Research Assessment Exercise would provide an incentive to do so [140]. While there is a choice of possible indicators of institutional interest and investment in public engagement and outreach activity, and adequate measures and proxies can be developed, they are of limited use in capturing the outcomes of such activities and their actual benefits for the populations concerned [112]. These limitations were powerfully stated in the report of a recent study of 15 European countries, which showed that “analysis and evaluation of PE [public engagement] activities is still underdeveloped, lacking robust and shared indicators of output and performance” [141]. Whereas standardised surveys can be used successfully to collect data about public engagement activity [118,140,141], there remains a challenge for HEFCE to develop rigorous methods to assess their quality and outcomes – both for the purpose of the REF and to support those universities who wish to promote it on the institutional level.

In Oxford, 54% of respondents to the impact assessment survey reported participating in patient and public involvement activities during the five-year census period. Although no direct comparison is possible, this may be lower than implied by national, multi-disciplinary studies. For example, the 2006 Royal Society survey found that 74% of scientists and engineers in various disciplines, including clinical medicine, had participated in science communication or public engagement activities in the year prior to the survey date [140], while a 2000 survey by the Wellcome Trust and MORI found that 52% of clinical biomedical scientists had participated in such activities in the previous year [118]. The patient and public involvement activities reported in this survey were very diverse in nature, including acting as a Science and Engineering Ambassador, giving public lectures and talks to patient groups, communicating with patients and clinical trials volunteers, acting as a patron to a patient support group, participating in the NIHR Biomedical Research Centre (BRC) Open Days, collaborating with a Wellcome Trust Artist-in-Residence, being interviewed by a journalist, maintaining a public website, engaging with policy-makers and NGOs, working with science centres and museums, organising workshops for pupils at local schools, and judging various competitions.

Given the many ways in which public engagement activity manifests itself, it is challenging to compare them in a standardised manner. For example, one respondent mentioned “public events to raise awareness of issues surrounding stem cell biology: World Economic Forum Annual Meeting, Davos…, [local schools], Witney.” How does researchers’ engagement with global leaders in Davos compare with their engagement with schoolchildren in the local community, and how does one estimate the value to the local community of world research leaders engaging with schoolchildren? Moreover, because the majority of respondents participated in several events every year the burden of collecting data about public engagement activity may be substantial. As one respondent – whose involvement with the British Science Association over several decades had led to many developments, including the creation of a science communication award – remarked:

“It is really too much to expect that one should keep a detailed account of all such activity and would indeed be inhibiting of such activity.”

In order to minimise the burden and standardise indicators, HEFCE can draw on the Wellcome Trust and Royal Society studies to develop a discipline-specific public engagement survey, which can be administered as part of the annual HEFCE Higher education-business and community interaction survey (HE-BCI).

### Improved social welfare, social cohesion or national security

#### Measures of improved social equity, inclusion or cohesion

There are several important ways in which universities can improve social equity, inclusion, and cohesion. Universities can enhance diversity and equality among their students and faculty. The percentage of women and ethnic minorities among applicants and matriculants to medical schools are duly regarded as key indicators in academic medicine [142,143]. In the UK specifically, the NIHR requires that universities applying for the next round of translational research funding hold at least the silver award of the Athena SWAN Charter for women in science [144]. Through a competitive application process, Athena SWAN confers bronze, silver and gold awards, which “recognise and celebrate good practice in recruiting, retaining and promoting women in STEMM [Science, Technology, Engineering, Medicine and Mathematics] in higher education” [145]. Nevertheless, such indicators of diversity, equality, and good practice are not regarded as impact indicators by HEFCE and thus were not included in the 2010 impact pilot. Traditionally, *inter alia*, such indicators are included in the “Environment” section of the RAE/REF, which will carry a weighting of 15% in the overall outcome of the REF. In countries with privately funded health care systems, medical schools and academic medical centres play an important role in improving social equity, inclusion, and cohesion by providing uncompensated health services to the populations most at risk of being underserved, i.e. the uninsured and members of disadvantaged communities [146]. Measuring the extent of such health services, both in terms of their monetary value and percentage in the overall provision of health services, would be an important indicator of societal and economic impact. In the UK, however, everyone has equal access to academic medicine because the National Health Service is publicly funded, and it is free at the point of use for everyone who is resident in the UK. Yet, there is some variation in the morbidity and mortality rates as well as in the provision and quality of services across the UK, which medical schools and university-employed clinical academics working in the NHS can address. For example, Oxford clinical academics contributed to the design and implementation of the National Stroke Strategy to reduce regional variations in the quality of stroke care and to the establishment of a new academically-led Stroke Unit in the Oxford John Radcliffe Hospital to improve stroke care in Oxford and Oxfordshire. Such contributions can be captured through the measures of improved health services and changes to public policy, as discussed above.

Whereas health is a universal human right [147], disadvantaged populations in less developed countries are trapped in the vicious circle of poverty, lack of education, social inequalities, tropical diseases, and poor health [148,149]. The responsibility of the international community to uphold human dignity, equality and equity is encompassed in the eight United Nations Millennium Development Goals (MDGs), each of which has its own targets, with measurable indicators [150]. Through its initiative “Academic Impact”, the United Nations seeks a commitment on the part of research-intensive institutions of higher education to “the fundamental precepts driving the United Nations mandate, in particular the realization of the universally determined Millennium Development Goals” [151]. There are four MDGs to which medical research can contribute by focusing on the health needs of disadvantaged populations in less developed countries:

• reduce child mortality;

• improve maternal health;

• combat HIV/AIDS, malaria, and other diseases; and

• develop a global partnership for development.

Research in tropical medicine and global health is the major avenue for clinical scientists in Oxford to contribute to the achievement of the MDGs. The university has tropical medicine research groups, who are permanently based in Wellcome Trust-Oxford University centres in Kenya, Thailand, Viet Nam, Laos, Tanzania, Indonesia and Nepal, as well as collaborators around the world [152]. Their activities range from basic, epidemiological and clinical research to behavioural sciences and use of public health evidence to monitor progress towards the MDGs [152]. Crucially, capacity building is integral to all tropical medicine and global health activities, and several respondents to the impact assessment survey emphasised that capacity building in developing countries should be included in impact indicators. As an example of their impact in sub-Saharan Africa, several respondents mentioned setting up clinical trials, purchasing high-specification equipment, large-scale long-term AIDS education and prevention programmes, training programmes for practitioners and researchers, and fundraising activities. Such activities provided unique resources to the region concerned and contributed to the development of specialised skills, as well as having had an educational impact on important segments of the population. It follows that in addition to the targets and measurable indicators encompassed in the MDGs, impact on health equality needs to include case studies focusing on research-based capacity building activities, such as mentoring the local academic and clinical workforce, developing local infrastructure, and contributing to education in local communities.

#### Application of new security technologies or practices

The UK National Security Strategy recognises that in today’s globalised world infectious diseases are among the major security challenges, and that the highest risk is an influenza-type pandemic [153]. It is estimated by the government that there is a high probability of such a pandemic occurring and that “possible impacts of a future pandemic could be that up to one half of the UK population becomes infected, resulting in between 50,000 and 750,000 deaths in the UK, with corresponding disruption to everyday life” [153]. In view of this, research-intensive universities and academic health centres have an important role in improving national and global security by addressing the risks of infectious diseases. In Oxford, examples of research-based activities aimed at addressing the risk of infectious diseases include the development and patenting of new technologies that subsequently were used by pharmaceutical companies for the preparation of H1N1 (swine flu) and H5N1 (bird flu) vaccines. In another example, following the declaration of a global H1N1 (swine flu) pandemic by the World Health Organization in June 2009, clinical scientists in Oxford, together with their colleagues in Bristol, Exeter, London, and Southampton, conducted the first trials of H1N1 vaccines in the UK and provided important information to the Department of Health to guide immunisation policy in the UK [154,155]. In order to assess the impact of such activities, both case studies and standardised measures of potential health outcomes can be used.

### Limitations

This article presents, to our best knowledge for the first time, empirical data to demonstrate how impact assessment operates and could operate in academic clinical medicine, providing funders and the medical research community with potential benchmarks for future comparative studies. However, our study has several methodological and data limitations, which entail the possibility of bias in the results. Administrative data for the proposed impact indicators were gathered retrospectively. If the university and faculty members were asked in advance to collect the types of data required for impact assessment, the scope and granularity of the data presented here are likely to have been greater. Moreover, there are limitations on generalising from a voluntary survey of a relatively small population. Although the achieved response rate of 48.1% was relatively good, and sample proportions by gender, career stage, and staff category were similar to the population surveyed, there were slight discrepancies between our sample and the population surveyed (Table 1). Therefore, we cannot rule out the possibility of self-selection bias. Finally, a compulsory census of the entire population of clinical academics and scientists working in the university and its partner hospitals would have probably yielded different results. Given that universities can select which and how many faculty members to include in their institutional submissions to the RAE/REF [156], not all eligible faculty members are usually submitted because universities try to maximise their funding by selecting more accomplished faculty members. The grounds for selection may differ between those faculty members whose individual publications are included in the research outputs submitted by the unit, and those whose work is referenced in the impact part of the submission [157]. For the purpose of the REF impact pilot exercise, we surveyed only those faculty members whose publication outputs were submitted to the 2008 RAE and thus were most likely to have their publications submitted to the 2014 REF. As demonstrated in Table 1, the survey population is skewed towards senior and mid-career university scientists. If we surveyed the entire population of clinical academics and scientists working in the university and its partner hospitals, we would have probably found more examples of impact, especially from clinical practice, which would have most likely been spread more evenly across the population.

### Implications for performance-based research funding systems

Notwithstanding methodological and data limitations, the results of our study demonstrate the range and significance of impacts in clinical medicine in a given university, and enable us to critically assess the “common menu” of impact indicators proposed by HEFCE in 2010. While the majority of the proposed indicators have some validity, there are significant challenges in refining the current indicators and methodologies in a number of ways.

First, the wide range and significance of impacts captured in our study suggest that a clearer conceptualisation and standardisation of impact measures is required in order to increase the validity and reliability of impact assessment. The issue of validity relates to the degree of certainty that the proposed indicators measure what they claim to measure, i.e. research impact. For example, it is not certain that participation on public policy committees or changes to legislation reflect one’s research activity rather than a sense of civic duty. The issue of reliability is concerned with the consistency of measurements. Without applying precise measures of impact and criteria for the attribution of impact to specific research activity across all universities, any impact assessment will be inconsistent and, thus, unreliable. While developing new standardised measures can be very costly and challenging, this can be mitigated by concentrating on those indicators that are already standardised, *e.g.* research income, patents, and clinical trials. The danger of doing so, however, is that such indicators have not been specifically designed for measuring impact and thus can only be used as proxy indicators, and that the probabilities of impact associated with such proxy indicators are currently unknown. As discussed in the case of research funding, although research funding competitively-awarded for the purpose of improved patient care is much more likely to lead to improved patient care in a relatively short period of time than non-competitively-awarded funding for basic science research, the probabilities of achieving impact within specified time horizons and how such probabilities vary between different medical research funders are currently unknown. Moreover, it should be kept in mind that an obvious limitation of any attempt to develop and use either direct or proxy impact indicators for high-stakes assessments of research is that their robustness as indicators is threatened once they become targets and benchmarks for performance (Goodhart's law).

Second, the aggregation of impacts is not yet possible because the majority of the indicators studied are not standardised. While the data collected using the current indicators provide evidence of the wide range and significance of impacts and can be used for descriptive case studies, we are not able to add the data collected from individual researchers and teams together to create an aggregate indicator for all faculty members in a given university and then compare it across universities. If impact assessment is to provide incentives for every faculty member to increase the economic and social impact of their research, then the impacts of the research of every faculty member must be counted in. Likewise, if impact assessment is to reward those universities that deliver more benefits to the economy and society, then we need to find a way to aggregate various impact indicators into the net indicator of impact in order to compare universities. The trusty method of traditional peer review will be stretched to the limit by the demand to weigh all the different types of evidence for each of the indicators, assess their quality, and produce consistent, meaningful aggregate profiles for each submitting institution.

Third, there is a challenge in choosing between quantitative and qualitative methodologies. Our study demonstrates that some of the current indicators lend themselves well to being quantified, but some others can be described only qualitatively. The advantage of using quantitative indicators is that they can be standardised and aggregated, allowing universities to use them on a continuous basis to track their impact, compare it with other universities, and recognise the contribution of every faculty member, of whatever scale. At the same time, developing valid and reliable quantitative indicators and then collecting data to operationalise them may be costly and time intensive. The advantage of using qualitative indicators and case studies is that there are important areas of impact that can be captured only qualitatively, and evaluating research impact through qualitative case studies is a relatively quick and cost-contained alternative to quantitative indicators. However, the emphasis on qualitative indicators would stretch traditional peer review further and concentrate on the most prominent examples of impact, overlooking more modest contributions.

Fourth, our study highlights the necessity to balance carefully incentives for basic science, translational research, and clinical work across impact indicators and between impact assessment and conventional research outputs because the rewards associated with impact assessment and even impact indicators themselves can modify the behaviour of individuals and universities. On the one hand, it takes considerably more time and effort to make basic science discoveries and design new interventions than to evaluate and bring them to market. Excessive emphasis on impact may give disproportionate rewards to those who evaluate interventions discovered by others and bring them to market, without giving full recognition to basic researchers, who invent and bring new interventions to clinical evaluation. On the other hand, bibliometrics and other conventional indicators of research output recognise the work of basic scientists who publish in journals with high impact factors and are part of large groups of authors, but may disadvantage academic physicians who devote a significant part of their time to clinical work and publish in specialty-specific journals.

Fifth, there is a need for clear, collaboratively-developed guidelines for university-based information systems and data collection procedures. The current university information systems and procedures were not specifically designed to provide certain types and levels of granularity of data required for impact assessment, and individual researchers often do not keep a record of events and facts that can be interpreted as impacts. As a result, collecting impact data retrospectively puts a strain on the scope and accuracy of impact indicators and presents a significant burden upon universities and their faculty. At the same time, in addition to HEFCE, a number of publicly-funded research councils and medical research charities have started asking universities and individual grant recipients to provide evidence of the economic and social impact of sponsored research. For these reasons, collaboration between HEFCE and other public funding agencies, medical research charities, universities, and the wider medical research community is required in order to develop and validate a common set of impact indicators with universal guidelines on data collection. This will raise the quality of impact assessment, reduce the duplication of effort, minimise the burden of collecting data for different types of impact indicators, and allow faculty to engage with patient populations and local communities more effectively.

Finally, our study suggests that there is a potential for impact indicators to be used for objectives other than the allocation of public research funds under the REF, i.e. medical schools can use impact indicators as part of their social accountability strategies. Although the allocation of UK government funding to universities on the basis of impact provides universities with incentives to increase their societal and economic impact, it is unlikely that these incentives alone can change the long-term behaviours of universities. On the one hand, these incentives may not be sufficiently high to change the long-term behaviour of universities in clinical medicine, because government funding is no longer the main source of research funding in clinical medicine and only a small proportion of such funding is proposed to be allocated on the basis of impact. It is also impossible to use legal mechanisms to enforce the accountability of universities and individual researchers for the receipt of research funding predicated on impact, because impact, as it is currently defined and measured, cannot be included in research contracts. Furthermore, universities are highly devolved institutions, to the effect that deans of medical schools or heads of academic departments have no direct hierarchical powers to hold individual researchers accountable for the actual impact of their research, which, according to the current definition, is expected to be achieved within ten years. On the other hand, there is growing consensus among medical schools around the world that they need to achieve and demonstrate a greater impact on the health needs of the populations and societies they serve as part of their social accountability project. This goal is consistent with the goal of the government to increase the economic and social impact of publicly-funded research through the REF. If universities, public funding agencies, medical research charities, and the wider medical research community collaboratively develop measurable impact indicators and methodologies that enable universities to claim, track and compare the impacts of their research in a transparent and rigorous manner on a continuous basis, universities can use impact assessment as part of their social accountability strategies. This way, the medical research community can adjust their long-term behaviour in line with the requirements of social accountability and, in doing so, once again prove to the public and taxpayers its right to professional autonomy and self-regulation.

## Conclusion

Overall, the evidence from our study supports the claim that assessing impact is feasible, but that current methodologies will need to be significantly improved before using measurable impact indicators as a basis on which to change the long-term behaviour of universities. The impact indicators and methods discussed in this paper can be used successfully to identify the many areas where impacts (in the REF sense) occur and the wide range of forms that they take, in order to build an evidence base for descriptive case studies of impact and impact statements as part of the REF peer review in 2014. However, a quinquennial peer review of descriptive case studies and statements, and the funding incentives associated with it, are unlikely to elicit and sustain change in the long-term behaviour of universities in the field of clinical medicine. To do so, universities would need to be able to claim, track and compare the impacts of their research in a transparent and rigorous manner on a continuous basis as part of their social accountability strategies. This is a different task, which would require further debate about impact assessment to move away from a quinquennial peer review of descriptive evidence to a continuous monitoring and analysis of measurable impact indicators. The latter would entail developing a set of valid and reliable indicators; robust methodologies for attributing and aggregating impact; carefully balanced incentives for basic science, translational research, and clinical work; and clear guidelines for universities. Given the scope and complexity of these tasks, universities and the wider medical research and academic community have an important role to play in shaping public understandings of research impacts, of the nature of evidence required to claim and assess impacts, and of the robustness of methodologies and indicators for impact assessment in the REF 2014 and beyond.

## Competing interests

The paper reports on data from the University of Oxford, of which all three authors are employees. AMB is Head of the Medical Sciences Division; PVO is a member of the Medical Sciences Division; AO is based in the Social Sciences Division.

## Authors’ contributions

PVO conceived the study jointly with AMB, designed the questionnaire, collected and anonymised the data, carried out the coding and quantitative analysis, reviewed the literature on specific indicators, anonymised data from the administrative survey after collection and prior to their release to AO, and drafted the first version of the manuscript. AO reviewed the coding, contributed to planning the paper and structuring the argument, critically reviewed the first draft and contributed to the writing of the second draft, and contributed insights from studies on research assessment, impact measurement, and higher education policy, including staffing. AMB had oversight of the design and data collection for the HEFCE pilot exercise, contributed to the design, planning and co-ordination of the study, and critically reviewed all versions of the manuscript. All authors read and approved the final version of the manuscript.

## Pre-publication history

The pre-publication history for this paper can be accessed here:

http://www.biomedcentral.com/1472-6963/12/478/prepub
